# Angiogenesis, Angiogenic Factors, and Interstitial Cystitis/Bladder Pain Syndrome (IC/BPS)

**DOI:** 10.1111/iju.70420

**Published:** 2026-06-16

**Authors:** Naoki Yoshimura, Akira Furuta, Yuki Tobisawa, Taro Igarashi, Tetsuichi Saito, Tomohiro Ueda

**Affiliations:** ^1^ Department of Urology University of Pittsburgh School of Medicine Pittsburgh Pennsylvania USA; ^2^ Department of Urology Jikei University School of Medicine Tokyo Japan; ^3^ Department of Urology Gifu University Graduate School of Medicine Gifu Japan; ^4^ Department of Urology Shinshu University School of Medicine Matsumoto Japan; ^5^ Department of Urology Ueda Clinic Kyoto Japan

**Keywords:** angiogenesis, angiogenic factor, bladder pain syndrome, Hunner lesion, interstitial cystitis

## Abstract

The etiology of IC/BPS is multifactorial; thus, pathophysiology‐based phenotyping is needed for tailored treatments of IC/BPS patients. In this regard, it is now generally agreed that, when focusing on inflammatory responses in the bladder mucosa, IC/BPS is divided into two major phenotypes. One is “interstitial cystitis (IC),” which is a chronic inflammatory disorder with Hunner lesions, and the other is “bladder pain syndrome (BPS),” in which inflammatory changes are minimal without Hunner lesions. However, as another important pathophysiological change in the IC/BPS bladder, angiogenetic responses including overexpression of angiogenic growth factors, such as platelet‐derived endothelial cell growth factor (PD‐ECGF), vascular endothelial growth factor (VEGF), and CD31 (platelet endothelial cell adhesion molecule; PECAM‐1), have been shown to correlate with symptom severity in Hunner‐type IC and non‐Hunner IC/BPS patients. In addition, previous studies in IC/BPS patients showed that glomerulations or mucosal bleeding during hydrodistension occur due to the rupture of bladder mucosal capillary vessels in association with PD‐ECGF and VEGF overexpression. Furthermore, upregulation of angiogenic factors and their signaling pathways has been detected in animal models of IC/BPS induced by bladder inflammation or psychological stress. Thus, angiogenic factor overexpression and immature microvessel formation in the bladder mucosa could be another important diagnostic criterion in addition to the inflammatory status for further phenotyping of IC/BPS.

**Abbreviations**
BPH: benign prostate hyperplasia
BPS: bladder pain symptoms
CD31: platelet endothelial cell adhesion molecule
DMSO: dimethyl sulfoxide
DRG: dorsal root ganglia
FGF: fibroblast growth factor
GAG: glycosaminoglycan
HIC: Hunner‐type interstitial cystitis
HIF: Hypoxia‐inducible factor
IC: interstitial cystitis
ICICJ: International Consultation on IC
MBAD: mucosal bleeding after distension
NBI: narrow‐band imaging
OAB: overactive bladder
PD‐ECGF: platelet‐derived endothelial cell growth factor
PDGF: platelet‐derived growth factor
TGF‐β: transforming growth factor‐β
TNF‐α: tissue necrotic factor‐α
TLR4: Toll‐like receptor 4
VEGF: vascular endothelial growth factor
VEGFR: VEGF receptor
WAS: water‐avoidance stress
WLI: white light imaging

## 1 Introduction

Interstitial cystitis/bladder pain syndrome (IC/BPS) is a chronic disease characterized by suprapubic pain and lower urinary tract symptoms. Because of the heterogeneous nature of this disease with its multifactorial etiology, clinical trials in the all‐inclusive populations of IC/BPS patients without phenotyping in the last decade have led mainly to failure to discover new therapeutic modalities of IC/BPS [1]. Thus, phenotyping IC/BPS, aimed at identifying bladder‐centric and/or outside‐the‐bladder pathologies, including cystoscopic observation of Hunner or non‐Hunner lesions of the bladder mucosa, is particularly important for the future of IC/BPS management. Based on recent discussions at international conferences including the 5th International Consultation on IC, Japan (ICICJ) meeting in 2024, it has been proposed that IC with Hunner lesion (Hunner‐type IC) should be separated from non‐Hunner IC/BPS because of the distinct inflammatory profiles and epithelial denudation compared with non‐Hunner IC/BPS.

In addition, in the 1988 NIDDK IC criteria, mucosal bleeding after hydrodistension (i.e., glomerulations) during cystoscopic examination was included as a diagnostic criterion of IC. However, glomerulations seen in IC/BPS patients do not correlate with symptoms and are also found in patients without IC/BPS [2]. Thus, IC/BPS has been subdivided into two main entities: Hunner‐type IC and non‐Hunner BPS regardless of the existence of glomerulations during bladder distension, as described in the recent IC/BPS guidelines including the East Asian guideline [3]. Nevertheless, previous studies have demonstrated that angiogenesis with neovascularization leads to the formation of fragile blood vessels in the bladder wall of IC/BPS, including the non‐Hunner subtype, and that these newly formed vessels are more susceptible to rupture, particularly during procedures like hydrodistension, resulting in the characteristic bleeding seen in glomerulations [4, 5]. Also, there has been clinical evidence showing that overexpression of angiogenic factors such as VEGF and PD‐ECGF is associated with more severe symptoms in IC/BPS patients [4, 6, 7]. Therefore, although glomerulations shown by mucosal bleeding upon bladder hydrodistension are not unique to IC/BPS [2], underlying microvessel formation in the IC/BPS bladder mucosa due to angiogenetic factor upregulation could be another important pathophysiology in both non‐Hunner IC/BPS and HIC in addition to significant inflammatory properties seen in Hunner type IC bladders. Thus, this review article summarizes clinical and basic research findings in angiogenesis‐related mechanisms underlying IC/BPS pathophysiology. In addition, because of some inconsistency in the IC/BPS nomenclature, this review used a phenotypic classification into three subtypes; (i) “Hunner‐type IC” that shows Hunner lesions, (ii) non‐Hunner IC/BPS that shows the hypervascular mucosa, and (iii) non‐Hunner BPS without apparent mucosal abnormalities.

## 2 Hunner Lesions and IC/Bps Phenotyping

Hunner‐type IC (HIC), which is the pathological entity distinct from no‐Hunner IC/BPS, is characterized by significant lymphocytic infiltration and epithelial denudation compared with non‐Hunner IC/BPS patients [8]. A Hunner lesion seen in HIC patients is not an “ulcer,” but rather a distinctive inflammatory lesion presenting as a reddened mucosal area with fragile microvessels radiating toward a central scar [1].

Based on active discussions on this topic at the ICICJ meetings including the 5th one in 2024, as well as the recent East Aian IC guideline [3], the current consensus is that Hunner‐type IC with significant inflammatory conditions in the bladder is clinically and pathologically distinct from non‐Hunner IC/BPS and categorized as a separate disease entity from other IC/BPS conditions without Hunner lesions [8, 9, 10]. The importance of IC/BPS phenotyping using cystoscopy‐based detection of Hunner lesions has been further supported by recent articles including those from North America [11, 12].

**FIGURE 1:**
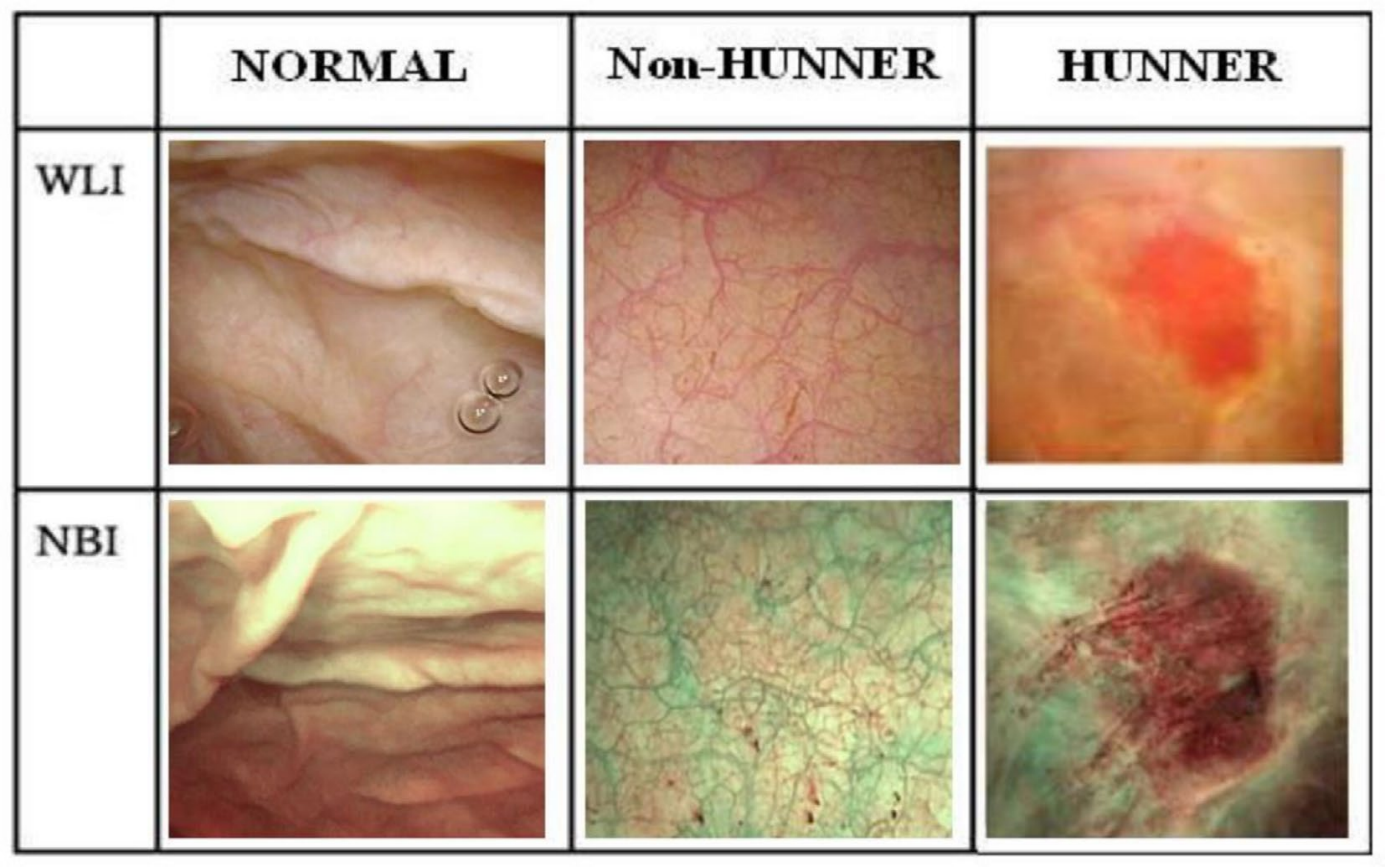
Photographs of cystoscopic images of normal bladder with mucosal folds and without apparent microvessel formation (left column), hypervascular, non‐Hunner bladder with mucosal microvessel formation (middle column), and Hunner‐type IC bladder with microvessel formation surrounding the Hunner lesion (right column). In each column, upper and lower rows of photos show the images taken at the same areas of the bladder under conventional white light (WLI) and narrow‐band imaging system (NBI), respectively.

Figure 1 shows the comparison of typical cystoscopic images of (i) normal bladder with mucosal folds and without apparent microvessel formation, (ii) hypervascular non‐Hunner IC/BPS bladder with mucosal microvessel formation, and (iii) Hunner‐type IC bladder with microvessel formation surrounding the Hunner lesion. In addition, by using the narrow‐band imaging (NBI) system, the bladder images more clearly showed microvessels in brown color within the superficial mucosal layer, which are distinctively different from the normal bladder with multiple mucosal folds and minimal superficial microvessel formation, compared to conventional white light images (WLI) (Figure 1) [13].

## 3 Glomerulation and Angiogenesis

**FIGURE 2:**
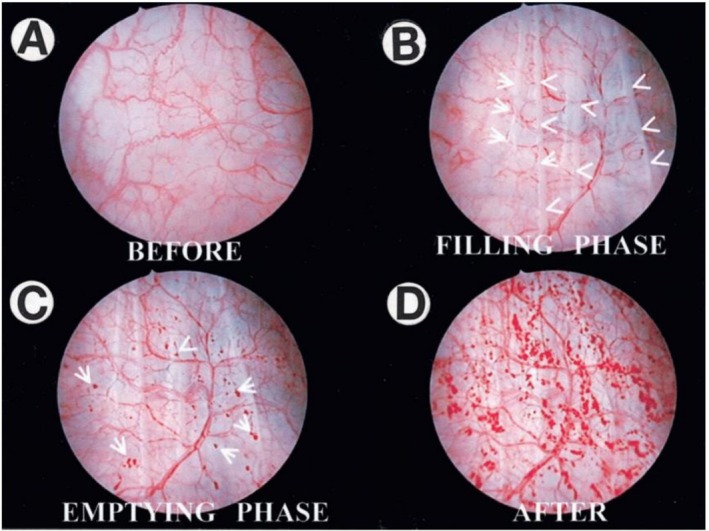
Cystoscopic views of glomerulations during bladder distension in a non‐Hunner IC/BPS patient. (A) Before distension; (B) Filling phase; (C) Emptying phase; (D) After distension. During bladder filling, whitish fibrous bundles emerged and interrupted blood flows of mucosal microvessels (arrowheads). During the emptying phase, petechial bleeding began from distal capillaries along fibrous bundles (arrowheads). Adopted from fig. 1 of Ref. [5].

Glomerulation or mucosal bleeding after distension (MBAD), which is characterized by pinpoint bleeding or small areas of hemorrhage on the bladder wall during cystoscopic procedures such as hydrodistension, has been recognized as an important clinical finding of IC/BPS (Figure 2) [5]. However, in contrast to Hunner lesions, it has been demonstrated that glomerulation is not specifically seen in non‐Hunner IC/BPS patients because it is also found in patients without IC/BPS, such as those with benign prostate hyperplasia (BPH) or urinary stones [2].

Previous research has indicated that glomerulations in IC bladders are associated with angiogenesis with the formation of fragile, immature blood vessels that are more prone to rupture. A previous study using cystoscopic observation confirmed that the blood flow in bladder wall vessels was interrupted by whitish fibrous bundles during bladder distention and then petechial bleeding, which defines glomerulations began from capillaries distal to obstructed vessels during bladder emptying (Figure 2) [5]. Taken together, these findings suggest a potential link between vascular changes and glomerulations in IC/BPS. Therefore, although glomerulations shown by mucosal bleeding upon bladder distension are not unique to IC/BPS [2], underlying vascular changes in the bladder mucosa due to angiogenesis could be an important pathophysiology in both non‐Hunner and Hunner‐type IC/BPS bladders, as also shown in other chronic inflammatory diseases such as diabetic retinopathy, atherosclerosis, and inflammatory bowel disease [14, 15].

In addition, there have been attempts to examine the relationship between the degree of glomerulations and bladder pathophysiologic properties in IC/BPS patients. For example, when glomerulation grade was classified into 0, none; 1, less than half of the bladder wall; 2, more than half of the bladder wall; or 3, severe waterfall bleeding during bladder distension, higher glomerulation grades were significantly associated with the reduced MBC as well as increased urinary levels of prostaglandin E2 (PGE2) and cytokines/chemokines such as CXCL10, MCP‐1, IL‐6, and RANTES in non‐Hunner IC/BPS patients [16, 17]. Also, another study by the same group of researchers reported that non‐Hunner IC/BPS bladders with grade 3 glomerulations showed similar levels of urothelial impairment to those detected in Hunner‐type IC bladders, evident as decreased and increased expressions of mature and immature epithelial cell markers, respectively [18]. These results suggest that non‐Hunner IC/BPS with grade 3 glomerulations that showed severer inflammatory responses might be a distinct clinical and pathophysiological entity compared to other types of non‐Hunner IC/BPS with lesser grades of glomerulations.

## 4 Mechanisms Underlying Angiogenesis in IC/BPS Bladders

**FIGURE 3:**
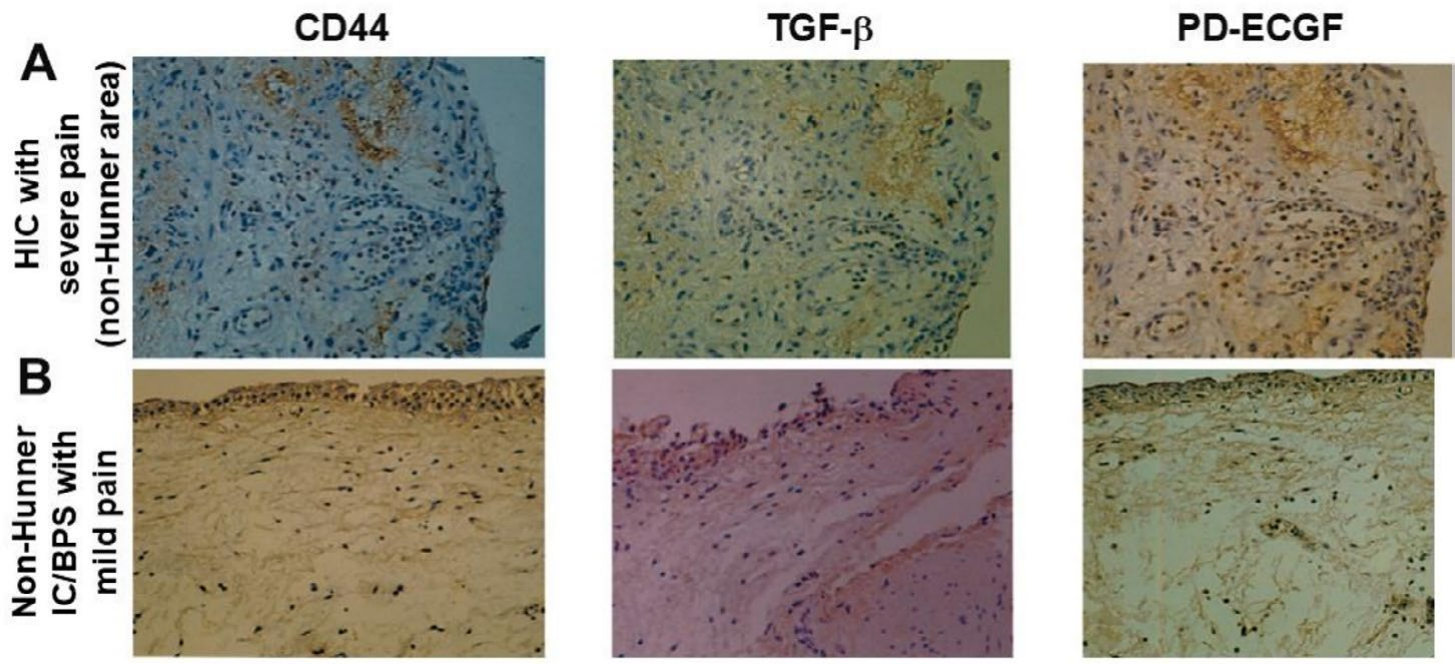
Photomicrographs of alternate bladder sections showing positive immunostaining of CD44, transforming growth factor‐β (TGF‐β), and PD‐ECGF in the bladder mucosal layer from a severe bladder pain patient with Hunner type (HIC) (A) and a mild bladder pain patient with non‐Hunner IC/BPS (B). While positive staining was observed along the basement urothelium membrane in patient B with bladder pain, positive staining was distributed at deeper mucosal areas in patient A with severe bladder pain. Reduced from X200. Modified from fig. 2 of Ref. [4].

**FIGURE 4:**
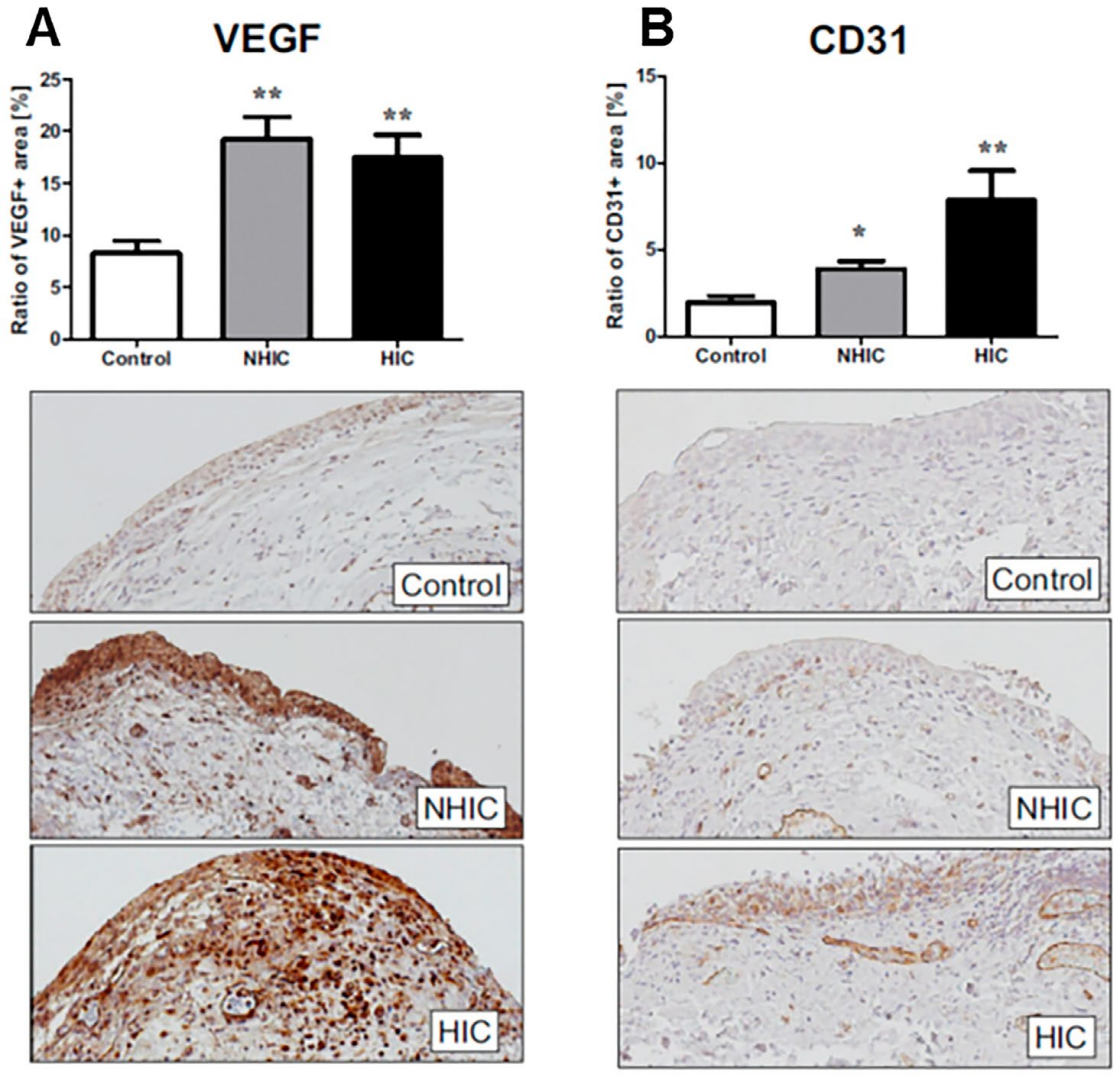
Bladder angiogenesis shown by the expression of VEGF (A) and CD31 (endothelial cell marker) (B). The expression of VEGF and CD31 was significantly increased in non‐Hunner IC/BPS (NHIC) and Hunner‐type IC (HIC) patients compared to controls. *p < 0.05; **p < 0.01 compared to controls. Adopted from fig. 2 of Ref. [7].

**FIGURE 5:**
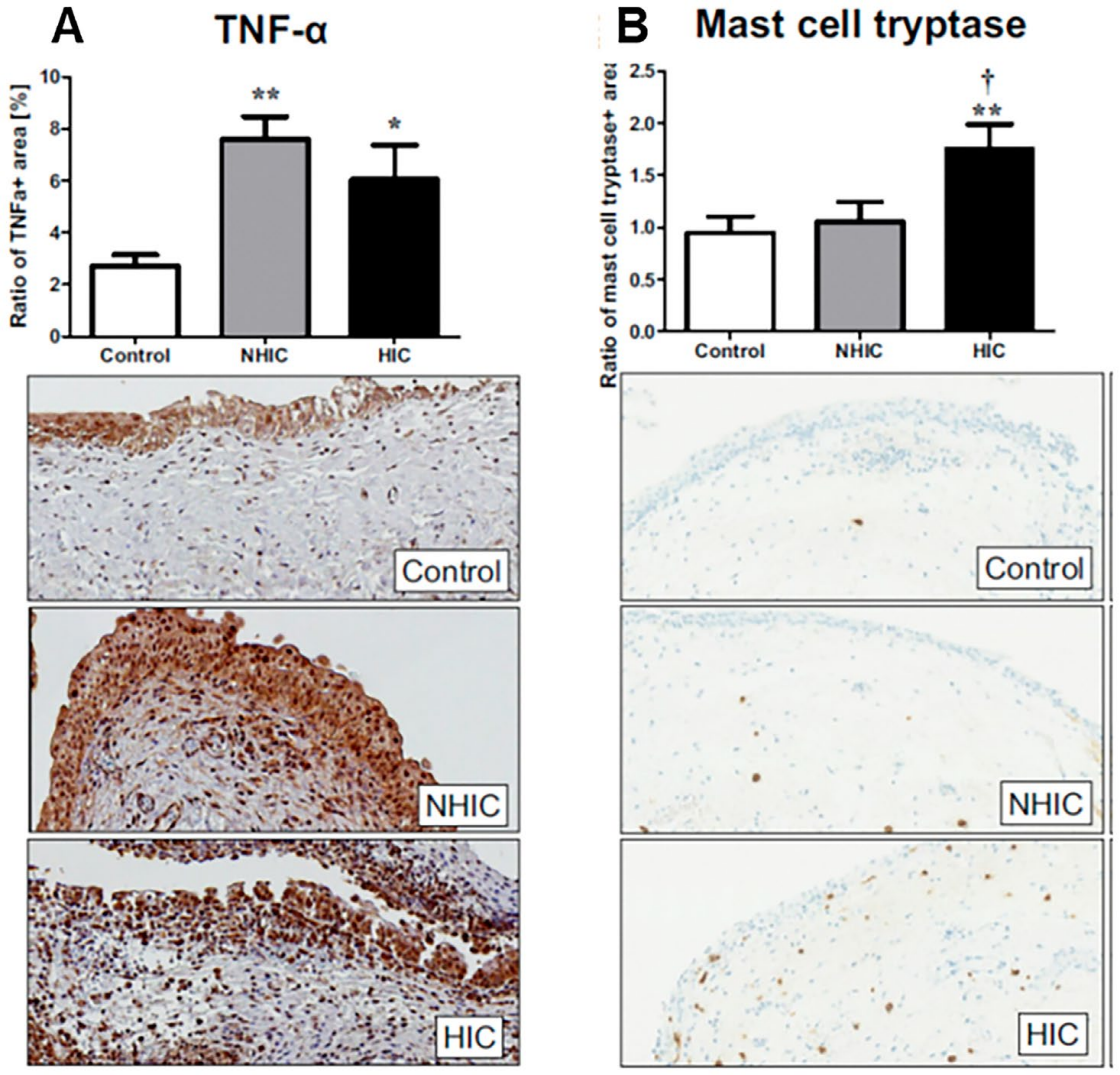
Bladder inflammation shown by the expression of TNF‐α (A) and mast cell tryptase (B). The expression of TNF‐α was significantly increased in both non‐Hunner IC/BPS (NHIC) and Hunner‐type IC (HIC) patients compared to controls, whereas a significant increase in the expression of mast cell tryptase was observed in HIC patients compared with control and NHIC patients. *p < 0.05; **p < 0.01 compared to controls, †*p* < 0.05 compared to NIHC. Adopted from fig. 1 of Ref. [7].

Angiogenesis is mediated by angiogenic factors that promote the formation of new blood vessels, and it is a vital process not only in normal physiological conditions such as wound healing but also in pathological conditions including cancer and diabetes [19]. Such angiogenic factors include vascular endothelial growth factor (VEGF), fibroblast growth factors (FGF), platelet‐derived growth factor (PDGF), and platelet‐derived endothelial cell growth factor (PD‐ECGF) [20]. In IC/BPS human specimens, overexpression of VEGF and PD‐ECGF has been found in the suburothelial layer (Figures 3 and 4) [5, 6, 7]. Also, previous studies using bladder tissues of patients with Hunner‐type IC or non‐Hunner IC/BPS have shown that (i) PD‐ECGF staining, which was observed in the mucosal layer beneath the urothelial basement membrane, was associated with immunostaining of transforming growth factor‐β (TGF‐β), an inflammatory cytokine (Figure 3) [5], and (ii) expressions of tissue necrotic factor‐α (TNF‐α) and TGF‐β were significantly increased in association with VEGF overexpression, although tissue infiltration of inflammatory cells such as mast cells, evident as a significant increased expression in mast cell tryptase, was only found in Hunner‐type IC, but not in non‐Hunner IC/BPS (Figure 5) [7]. Moreover, in other areas of research, TGF‐β reportedly enhanced the induction of VEGF in epithelial cells and fibroblasts to promote angiogenesis [21], and TNF‐α stimulation was shown to significantly increase VEGF mRNA and protein expression in fibroblast‐like synoviocytes of rheumatoid arthritis patients [22]. Thus, it is reasonable to assume that, as part of angiogenesis‐inducing mechanisms, production of inflammatory cytokines including TNF‐α and TGF‐β is closely interrelated with overexpression of angiogenetic factors (e.g., VEGF and PD‐ECGF) in IC/BPS bladders.

In addition, previous studies have shown that blood perfusion in the bladder was decreased in IC/BPS bladders, especially during the filling phase [23, 24] and that the expressions of hypoxia‐inducible factor‐1α (HIF‐1α) and VEGF proteins were both increased at the site of glomerulations in IC/BPS bladders compared to the control group [25]. HIF‐1α has been known to be a pivotal transcription factor induced under hypoxic response, leading to transactivation of target genes such as VEGF [26]. In addition, HIF‐1α mRNA and protein expressions were found to be increased in Hunner vs. non‐Hunner areas in Hunner‐type IC bladders [27]. Thus, it is likely that ischemia‐induced hypoxia due to the decreased blood perfusion in the IC/BPS bladder wall may contribute to upregulation of angiogenic factors such as VEGF, resulting in formation of immature microvessels, the rupture of which causes glomerulations during bladder distension in both Hunner‐type IC and non‐Hunner IC/BPS [5].

However, besides the involvement of inflammatory cytokines and/or hypoxia‐induced HIF‐1 induction, the underlying mechanisms inducing angiogenesis and angiogenetic factor upregulation in IC/BPS bladders are not fully elucidated. In this regard, our previous study has shown the increased immunostaining of CD44 in the suburothelial layer, where PD‐ECGF and TGF‐β were also stained, in Hunner IC and non‐Hunner IC/BPS bladders (Figure 3) [4]. CD44 is a cell surface receptor for hyaluronic acid, one of glycosaminoglycans (GAGs) that are a family of linear, highly negatively charged polydisperse polysaccharides [28], and expressed ubiquitously on the cell surface and in the extracellular matrix of various tissues [29]. It has been documented that the disruption in the GAG layer, which is a protective factor of the bladder urothelium, is one of the important pathophysiological etiologies of IC/BPS as the intravesical replenishing therapy of GAGs including hyaluronic acid is reportedly effective for the treatment of IC/BPS patients [30]. Previous studies have also shown that, when hyaluronic acid binds to CD44, it can enhance the production of VEGF, which promotes angiogenesis, leading to increased endothelial cell proliferation and migration—essential processes for new blood vessel formation [29]. Moreover, when binding to CD44, partially degraded, low‐molecular‐weight oligosaccharide products of hyaluronic acid, produced by the action of degradation enzymes called hyaluronidases, rather than naïve, high‐molecular‐weight hyaluronic acid, are reportedly involved in the formation of new blood vessels in pathological conditions including vascular diseases and cancer development [29]. Also, hyaluronic acid biosynthesis plays an important role in cardiovascular development [31]. Hyaluronic acid is produced as a large polymer, but its metabolic rate is so rapid that 1/3 of it is replaced per day [32]. In 2017, Tobisawa et al., identified Transmembrane protein‐2, a key extracellular hyaluronic acid‐degrading enzyme responsible for the initial steps of this degradation mechanism [33] and reported that it is essential for hyaluronic acid metabolism in vivo [34]. This degrading enzyme molecule has been reported to regulate both cardiovascular formation and neovascularization in zebrafish [35, 36, 37]. In other words, the cycle of hyaluronic acid synthesis and metabolism, which is thought to exist randomly in the extracellular space, is very important for angiogenesis. Furthermore, it has been reported that low‐molecular‐weight hyaluronic acid may induce activation of the innate immune system, including modulation of dendritic cell migration and subsequent allergic responses via Toll‐like receptor 4 (TLR4) stimulation [38]. Inflammation resulting from this activation mechanism can injure nearby vascular endothelial cells, leading to elevated HIF‐1α levels and increased production of VEGF [39].

Thus, it could be hypothesized that degraded GAGs including low‐molecular‐weight hyaluronic acid, which is produced by hyaluronidases including Transmembrane protein‐2, bind to CD44, whose expression is increased in IC/BPS, and enhance the production of VEGF, leading to angiogenesis with new microvessel formation and glomerulations induced by microvascular rupture during bladder distension in IC/BPS bladders. Further studies are warranted to investigate pathophysiological changes in the pathways of hyaluronic acid–hyaluronidase–CD44–angiogenic factor interactions in the context of IC/BPS pathogenesis.

## 5 Angiogenesis and IC/BPS Symptom Severity

There has been clinical evidence showing the correlation of IC/BPS symptom severity and the degree of angiogenesis revealed by angiogenic factor overexpression or new blood vessel formation in IC/BPS bladders.

Ueda et al. have compared the PD‐ECGF expression of IC/BPS bladder mucosa between severe‐ and mild‐pain patient groups and observed that six IC patients with severe pain (5 Hunner type and 1 non‐Hunner cases) had PD‐ECGF immunostaining in deeper suburothelial levels and higher protein concentrations of PD‐ECGF (*p* < 0.01) in mucosal biopsy specimens compared to thirteen IC patients with mild pain (1 Hunner type and 12 non‐Hunner cases) or control patients without pain [4] (Figure 3). Also, Kikuchi et al. revealed in IC/BPS patients with glomerulations during bladder distension that VEGF expression in the suburothelial layer was significantly higher in IC/BPS than in control samples (50% vs. 10%, *p* < 0.05).and that, among patients with IC/BPS, VEGF expression was significantly higher in those with severe pain than in those with mild pain (78% vs. 38%, *p* < 0.05). They also showed that the microvessel pericyte coverage index (MPI), which is a marker of mature vessel formation, was significantly lower in IC/BPS than in control samples (23% vs. 35%, *p* < 0.05), indicating the formation of immature vessels, which could contribute to glomerulation during bladder distension, in IC/BPS bladders [6]. Furthermore, a more recent study by Furuta et al. demonstrated that VEGF immunostaining areas in biopsied mucosal tissues were significantly (*p* < 0.01) increased in both non‐Hunner IC/BPS and Hunner‐type IC patients at similar levels compared to non‐IC/BPS controls [7] (Figure 4). They also found that suburothelial expression areas of CD31, which is platelet endothelial cell adhesion molecule/PECAM‐1 and a marker of endothelial cells that can be used to evaluate the degree of angiogenesis, were significantly increased in both non‐Hunner IC/BPS (*p* < 0.05) and Hunner‐type IC patients (*p* < 0.01) compared to non‐IC/BPS controls (Figure 4) and that angiogenesis evident as increased CD31 expression is strongly correlated with O'Leary‐Sant symptom and problem indexes and visual analog scale pain scores in IC/BPS patients (7). Also, another study by Akiyama et al. showed that VEGF and CD31 expressions in biopsied mucosal specimens from Hunner‐type IC patients were significantly increased compared to those from non‐Hunner IC/BPS patients, although VEGF or CD31 expression was not different between non‐Hunner IC/BPS and non‐IC/BPS patients in their study [40].

In addition, it has been reported that VEGF is one of the significant urine markers that can discriminate IC/BPS patients (both Hunner IC and non‐Hunner IC/BPS) from overactive bladder (OAB) patients [41, 42] and that urinary symptoms and pain severity were significantly correlated with urinary VEGF levels in female IC/BPS patients [43]. Taken together, these results indicate that angiogenesis with angiogenic factor overexpression and immature microvessel formation, which are the underlying conditions of glomerulations during bladder distension, are the important pathophysiological manifestations that are correlated with bladder pain and other symptoms of IC/BPS including non‐Hunner IC/BPS subtype.

Finally, angiogenesis with increased VEGF and HIF‐1α expression has been detected in bladder specimens from BPH patients with bladder outlet obstruction [44]. Also, glomerulations resulted from angiogenetic changes with microvessel formation are reportedly nonspecific for IC/BPS bladders [2]. Thus, the detection of bladder angiogenesis or glomerulation is not enough for the diagnosis of IC/BPS, especially for the non‐Hunner type. In this regard, a recent clinical trial of dimethyl sulfoxide (DMSO) has utilized intravesical lidocaine instillation to confirm that their bladder abnormalities, either Hunner lesions or glomerulations, were a source of bladder pain in IC/BPS patients [45]. Thus, the three‐step approach consisting of (i) the presence of bladder‐related pain symptoms, (ii) detection of bladder pathologies by cystoscopic examination, and (iii) verification that bladder pathological changes including angiogenesis are the causes of patients' symptoms has been proposed as the Ueda Criteria for the proper diagnosis of bladder‐centric IC/BPS [1, 46].

## 6 Angiogenic Mechanisms in Animal Models

Because IC/BPS etiology is thought to be multifactorial including inflammatory, neurogenic, autoimmune, and/or vascular abnormalities [1], basic research using animal experimental models that mimic IC/BPS, at least some aspects, has been performed to better understand the IC/BPS pathophysiology. Each of the models usually focuses on a certain system to recreate bladder dysfunction, which is useful to help understand each of the contributing pathophysiological mechanisms [47, 48]. In this regard, there have been basic research studies focusing on angiogenesis and angiogenetic factor expression in animal models of bladder inflammation or psychological stress. For example, in mice, intravesical instillation of VEGF induced bladder overactivity and pelvic hypersensitivity in association with upregulation of C‐fiber afferent markers such as TRPV1 and TRPA1 in lumbosacral dorsal root ganglia (DRG), suggesting that there is a direct interaction between VEGF and pelvic sensory pathways to induce IC/BPS‐like symptoms [49, 50]. Also, in a rat model of bladder inflammation induced by intravesical hydrochloric acid instillation, oral administration of axitinib, an anti‐angiogenic tyrosine kinase inhibitor, for 5 days decreased bladder overactivity and paw pain sensitivity with reductions in angiogenesis and VEGF/PDGF receptor expression in the bladder mucosa [51]. Furthermore, treatments with systemic application of anti‐VEGF antibodies or intravesical instillation of a VEGF receptor 2 (VEGFR2) tyrosine kinase inhibitor ameliorated bladder overactivity and pelvic hypersensitivity in rodent models of bladder inflammation induced by cyclophosphamide [52, 53]. In addition, a recent study reported that psychological stress model rats induced by water‐avoidance stress (WAS) showed increased microvasculature formation in association with VEGF upregulation in the bladder mucosa [54]. It has been reported that WAS‐elicited psychological stress induced not only various central effects but also local effects including urothelial damage, which may become a preceding bladder condition for further bladder insults to induce bladder overactivity and pelvic pain [55, 56]. Thus, it is assumed that these stress‐mediated local effects could contribute to angiogenesis and bladder inflammatory responses in animal model bladders. Taken together, it is assumed that anti‐angiogenetic therapies have a high potential for the treatment of IC/BPS patients.

## 7 Conclusion

**FIGURE 6:**
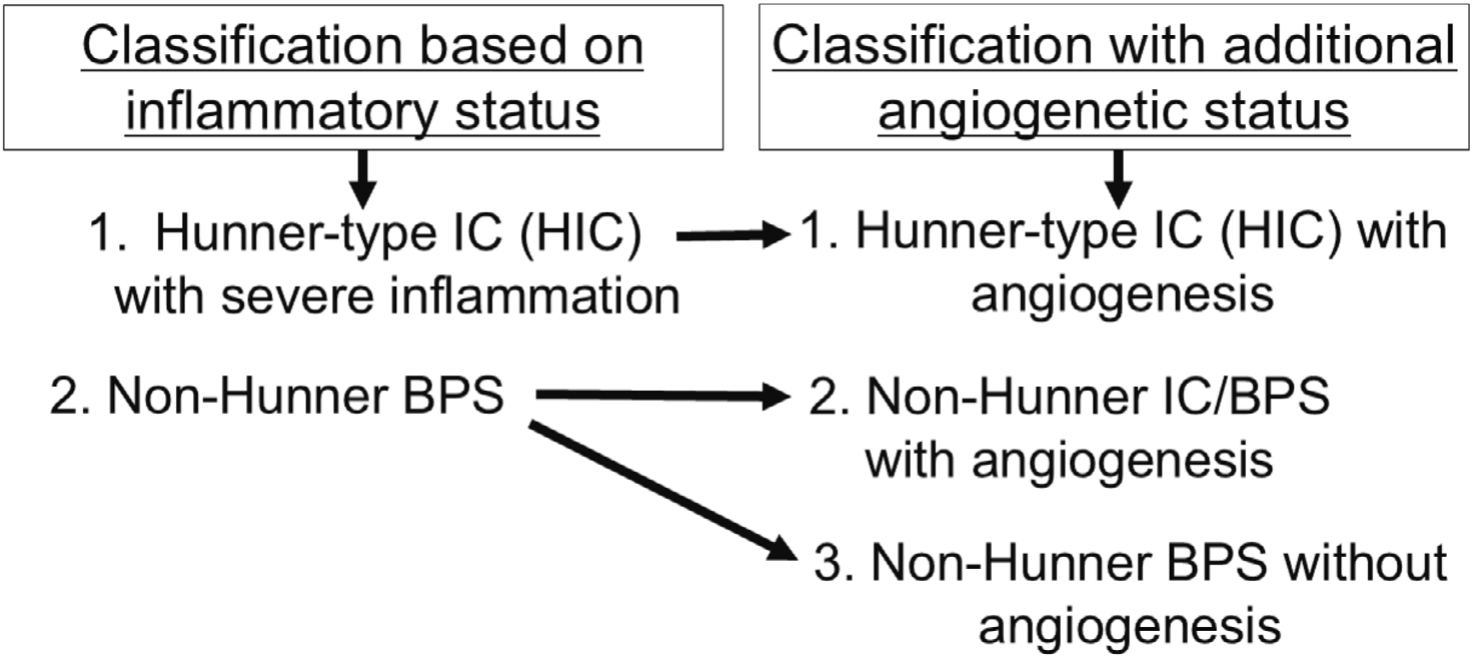
Proposal for a new classification of IC/BPS based on inflammatory and angiogenic status in the bladder.

The recent major advance in the clinical and basic research field of IC/BPS is the characterization of Hunner‐type IC that shows distinct immunogenic and inflammatory characteristics, different from other IC/BPS conditions with much lesser degrees of inflammatory responses. Thus, it has been proposed to subdivide IC/BPS into two entities, namely, inflammation‐prone Hunner‐type IC (HIC) and less‐inflammatory non‐Hunner BPS based on their inflammatory status [3]. However, as summarized in this review, angiogenesis with immature microvessel formation and angiogenic factor upregulation in the bladder, which are correlated with IC/BPS symptom severity, seem to be important pathophysiologic factors involved in HIC as well as non‐Hunner IC/BPS with hypervascular mucosa. Thus, by taking not only inflammatory aspects but also angiogenetic characteristics into account as the combined diagnostic criteria, IC/BPS could be further classified into (i) Hunner‐type IC with strong inflammatory and angiogenetic changes, (ii) non‐Hunner IC with angiogenesis/angiogenetic factor upregulation, and (iii) BPS without local inflammation or angiogenetic changes (Figure 6). Moreover, although glomerulation is a phenotypic manifestation of angiogenesis, revealed by distension‐induced rupture of immature microvessels, it has been shown to be nonspecific for IC/BPS diagnosis [2]. Therefore, further studies are much needed to develop more reliable, less‐invasive methodologies for evaluating angiogenetic changes in the bladder mucosa, instead of using glomerulations as a diagnostic criterion, for the future development of angiogenesis‐including classification and angiogenesis‐targeting therapies of IC/BPS.

## Author Contributions

Naoki Yoshimura: Writing – original draft; writing – review and editing; project administration. Akira Furuta, Tetsuichi Saito, Yuki Tobisawa, Taro Igarashi: Writing – original draft – review and editing. Tomohiro Ueda: Project administration.

## Conflicts of Interest

The authors declare no conflicts of interest.
